# An In Vivo Model of Estrogen Supplementation Concerning the Expression of Ca^2+^-Dependent Exchangers and Mortality, Vitality and Survival After Myocardial Infarction in Ovariectomized Rats

**DOI:** 10.3390/jcdd11110352

**Published:** 2024-11-02

**Authors:** Tomáš Toporcer, Tomáš Grendel, Ivana Špaková, Alžbeta Blichárová, Ľudmila Verbóová, Zuzana Benetinová, Beata Čižmárová, Miroslava Rabajdová, Silvia Toporcerová

**Affiliations:** 1 Department of Heart Surgery, East Slovak Institute of Cardiovascular Disease and Faculty of Medicine, Pavol Jozef Šafárik University, 040 11 Košice, Slovakia; topyto@gmail.com; 2 Department of Anesthesiology and Intensive Medicine, East Slovak Institute of Cardiovascular Disease and Faculty of Medicine, Pavol Jozef Šafárik University, 040 11 Košice, Slovakia; 3 Department of Medical and Clinical Biochemistry, Faculty of Medicine, Pavol Jozef Šafárik University, 040 11 Košice, Slovakiabeata.cizmarova@upjs.sk (B.Č.); miroslava.rabajdova@upjs.sk (M.R.); 4 Department of Pathology, Louis Pasteur University Hospital and Faculty of Medicine, Pavol Jozef Šafárik University, 040 01 Košice, Slovakia; alzbeta.blicharova@upjs.sk (A.B.); ludmila.verboova@upjs.sk (Ľ.V.); zuzana.benetinova@upjs.sk (Z.B.); 5 Department of Gynecology and Obstetrics, Faculty of Medicine, Pavol Jozef Šafárik University and Gyncare, 040 11 Košice, Slovakia

**Keywords:** myocardial infarction, rhythm disorders, estrogen supplementation, *NCX1*, ovariectomized rat

## Abstract

Background: Ischemic-reperfusion damage of cardiomyocytes due to myocardial infarction (MI) often leads to the death of an individual. Premenopausal women have been observed to have a significantly lower risk of cardiovascular disease (CVD) than men of the same age. In menopausal women, this trend is significantly reversed, and the risk of CVD increases up to 10-fold. Estrogens affect the development and function of the heart muscle, and as they decrease, the risk and poor prognosis of CVD increase. This study is focused on the effects of estrogen supplementation on morbidity, vitality, and *NCX1* expression after MI on a model system. Methods: In this study, female Sprague Dawley rats (n = 58), which were divided into three experimental groups (NN—control group, non-supplemented; OVX-N—ovariectomized, non-supplemented; OVX-S—ovariectomized, supplemented), received left thoracotomy in the fourth intercostal space. The left anterior descendent coronary artery was ligated 2 mm from its origin with an 8.0 suture. An immunohistological analysis as well as an RT-PCR analysis of *NCX1* expression were performed. Results: A higher survival rate was recorded in the OVX-N group (86%) in comparison with the OVX-S group (53%) (*p* < 0.05). In addition, higher *NCX1* expression 7 days/14 days after MI in the OVX-S group in comparison with the NN and OVX-N (*p* < 0.001 and *p* < 0.05) groups was recorded. Seven days after MI, a significantly higher expression (*p* < 0.005) of mRNA *NCX1* in the OVX-N group was also recorded in comparison with the NN group. Conclusions: This study provides a comprehensive description of the effect of estrogen supplementation on *NCX1* expression and overall vitality in ovariectomized rats that survived MI.

## 1. Introduction

Sex hormones regulate more than just the development and function of the sex organs; they are also involved in controlling the development of many other tissues, leading to slight differences in the size or physiological function of tissues or the microbiome between the sexes [1,2,3,4,5]. This gender difference is marked in cardiomyocytes, as well [6]. Heart tissue is rich in mitochondria [7], which are inherited exclusively on the maternal side [8]. The fact that women have fewer but more efficient mitochondria compared to men [9] could result from naturally higher levels of female estrogens, which are secreted in the ovaries and in the corpus luteum on the stimulus of androgens from the pituitary gland [10]. Estrogens can induce gene transcription of the mitochondria-encoded genes required for oxidative phosphorylation and other mitochondrial metabolic pathways [9]. At the same time, estrogens increase oxidative capacity, thereby reducing oxidative stress, via binding to G protein-coupled estrogen receptor (GPER), which subsequently activates antioxidant responses [11,12].

Effective mitochondrial metabolism not only refers to the generation of energy in the form of ATP but also participates in the intracellular calcium signaling [13] necessary for muscle contraction and other functions. In addition to their well-known antioxidant protective role, estrogens also affect the electrophysiological maturation of cardiomyocytes (prolonged action potential) as calcium-dependent proteins, such as sarcoplasmic/endoplasmic reticulum Ca^2+^-ATPase 2a (SERCA2a) [14] or sodium–calcium exchanger1 (*NCX1*), which is more expressed in women compared to men [15,16]. Studies on ovariectomized (OVX) rats showed a reduced expression of calcium-handling proteins, of which mainly *NCX1* is strongly regulated by the level of estrogens [9]. However, neither a positive nor negative effect of estrogens on the contractile function of the heart emerges from previous studies [17,18,19,20,21].

The adult heart has a limited regenerative capacity, while in general, women of reproductive age have a demonstrably better regenerative capacity of cardiomyocytes than men [22,23,24]. Reduced regenerative capacity of cardiomyocytes leads to increased deaths due to ischemic heart diseases, as cardiomyocytes suffer from ischemia-reperfusion injury [9]. Due to the higher level of estrogens, women have a better antioxidant defense mechanism; therefore, there is a significantly lower incidence of ischemic heart diseases in women with a physiological level of estrogens [25,26]. This fact changes with the onset of the postmenopausal period, and the risk of heart disease increases rapidly [27,28]. The same trend was also observed in patients who underwent ovariectomy [29]. These risk-increasing factors of CVD are more important in early or surgical menopause.

The heart is not only the target organ for the action of a number of hormones but also participates in the autocrine and paracrine production of hormones such as ANP (arterial natriuretic peptide), BNP (brain natriuretic peptide), endothelin1, vasostatin1, fibroblast growth factor 2, cardiac-derived oxytocin and others [9,30,31,32,33,34]. The hormones ANP and BNP are stored in secretory granules; they circulate freely, and by binding to natriuretic peptide receptors (NPRs), can activate the guanylate cyclase domain in NPR1/2, which increases intracellular cyclic guanosine monophosphate (cGMP), stimulates PKG (cGMP-dependent protein kinase) and, in addition, phosphorylates phospholamban (Plb), thereby activating the associated calcium-ATPase SERCA and enforcing calcium sequestration [35]. Subsequently, PKG phosphorylates the inositol triphosphate (IP3) receptor-associated PKG-1 substrate (IRAG) on the sarcoplasmic reticulum [36], which inhibits the leaching of calcium cations (Ca^2+^) through the IP3 receptor I (IP3RI) [37,38]. At the cellular level, cGMP-PKG signaling controls relaxation and contraction, as well as hypertrophy and apoptosis [9].

Strictly regulated calcium homeostasis is required for the physiological function of the heart muscle. Repolarization of the membrane potential to almost zero voltage triggers the opening of the LTCC in the plasma membrane of cardiomyocytes and allows the release of a small number of calcium cations in the ventricle [39]. This phenomenon leads to the opening of ryanodine receptors (RyRs) on the sarcoplasmic reticulum membrane, followed by the release of a large amount of Ca^2+^ and, in conclusion, rapidly increases its intracellular concentration [40,41]. Ca^2+^ binds to troponin C and triggers coarse/thin filament slip, leading to cardiomyocyte contraction [42]. The relaxation of cardiomyocytes is ensured by the reverse flow of Ca^2+^ into SR via SERCA, or Ca^2+^ is pumped out of the cell via the *NCX1* [27,42].

As estrogens decrease, *NCX1* expression also decreases; the SR Ca^2+^ overloads and increases myocardial contraction, and Ca^2+^ transient amplitude occurs [27]. The binding of E2 (estradiol) to the ER (estrogen receptor) reduces Ca^2+^ transient amplitude in ventricular cardiomyocytes stimulated by the increased concentration of catecholamines and restores cardiac contractility, not only under physiological conditions but also in the case of estrogen supplementation observed in OVX rats [27].

Hormone replacement therapy (HRT) is a possible therapy for avoiding negative changes in CVD development. While observational studies have suggested a reduced risk of cardiovascular disease with HRT, a randomized controlled trial conducted by the Women’s Health Initiative reported an increase in cardiovascular diseases as well as venous thromboembolism and breast cancer [29]. More detailed analyses point to the conclusion of a predominantly positive effect of early HRT, especially in young women and women with surgical menopause, which correlates with some guidelines [29,43]. Thus, if the effect of HRT on cardiovascular disease is ambiguous, the question arises of the effect of estrogens on the course and consequences of myocardial infarction (MI), should it occur.

This study aimed to determine the effect of estrogen supplementation on survival after MI in ovariectomized rats.

## 2. Materials and Methods

### 2.1. Animal Model

Female Sprague Dawley rats (n = 58), six months of age, were used in this study. These were randomly divided into three groups: a control group, not ovariectomized (NN) (n = 14); an ovariectomized group, not supplemented (OVX-N) (n = 14); and an ovariectomized and supplemented group (OVX-S) (n = 30).

**Figure 1:**
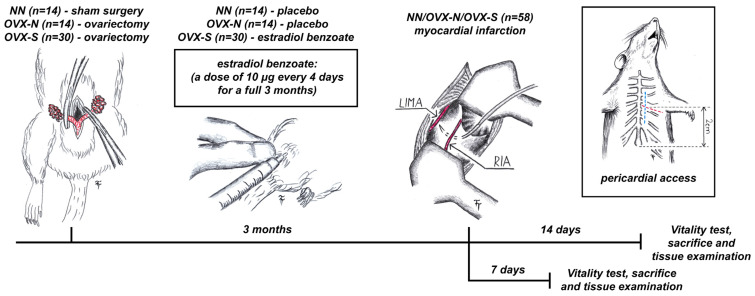
A diagram of the experiment design (blue dashed line—line of skin incision; red dashed line—surgical approach through the intercostal space).

Three months before the experiment, the animals from the OVX-N and OVX-S groups underwent an ovariectomy. The surgery was performed under inhalation anesthesia (semi-closed circuit) with isoflurane. Tramadol was injected intramuscularly in all the rats at a dose of 0.5 mg/kg. Atropine was administered subcutaneously as premedication at a dose of 0.05 mg/kg. In both groups, both ovaries were surgically removed. Animals from the NN group received a sham laparotomy without surgery on the ovaries. From the day of the ovariectomy to the myocardial infarction, animals from the OVX-S group received estradiol benzoate (Agofollin Depot, Switzerland) at a dose of 10 μg/animal every fourth day intramuscularly, as previously described [44,45]. The dosage was chosen to mimic the rat estrous cycle. Animals from the NN and OVX-N groups received an appropriate dose of isotonic solution in the same period (Figure 1).

### 2.2. Induction of Myocardial Infarction

**Figure 2:**
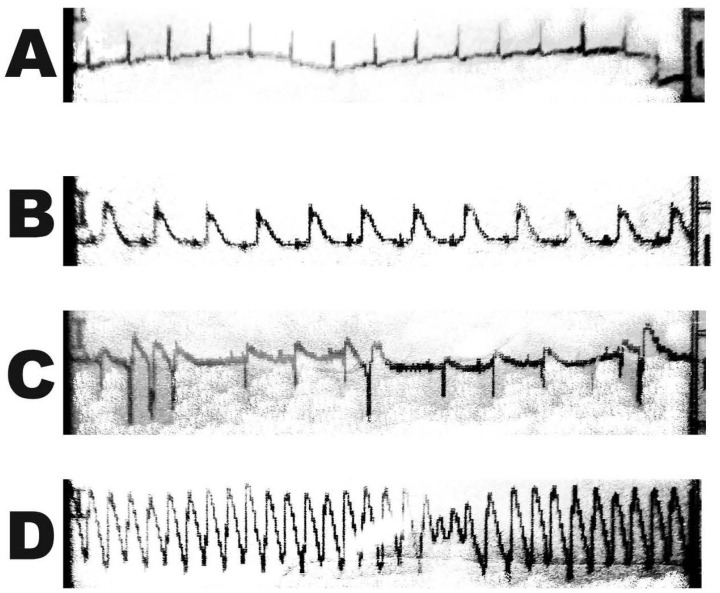
ECG curve during myocardial infarction: (**A**)—physiological ECG before the surgery; (**B**–**D**)—ECG changes after left anterior descending coronary artery ligation; (**B**)—ST segment elevation; (**C**)—ST segment elevation with ventricle arrythmias; (**D**)—ventricular tachycardia.

The animals from each group received a left thoracotomy in the fourth intercostal space. The left anterior descendent coronary artery (LAD) was ligated 2 mm from its origin with an 8.0 suture. MI was confirmed by electrocardiography (ECG) and discoloration of the myocardium distally to the ligated artery (Figure 2). Surgery was performed under inhalation anesthesia (semi-closed circuit) with isoflurane after orotracheal intubation. The same dose of tramadol and atropine as in the first surgery was used. The thorax was closured with a pleural drain, which was extracted after pneumothorax aspiration at the end of the anesthesia.

The mortality rate during MI in each group was recorded. After MI, the animals from each group were randomized into subgroups (_7 and _14) adequately according to mortality during MI in the individual group. Animals of subgroups NN_7, OVX-N_7, and OVX-S_7 were killed 7 days after MI. Animals of sub-groups NN_14, OVX-N_14, and OVX-S_14 were killed 14 days after MI.

### 2.3. Vitality Test—Graded Treadmill Run

Seven days (groups NN_7, OVX-N_7, and OVX-S_7) or fourteen days (groups NN_14, OVX-N_14, and OVX-S_14) after MI, the rats performed a graded treadmill run to fatigue on a customized rodent treadmill. The protocol involved the rats running in three stages, with a progressive increase in the treadmill speed: (1) 8 m/min, (2) 12 m/min, and (3) 18 m/min. The two initial stages lasted for 3 min each, whereas rats continued running in the final stage until they reached the point of fatigue, which was confirmed by the loss of the animal righting reflex. The test was described and used by other authors [46]. Subsequently, the total running distance was obtained. After the running test, the animals were killed and the hearts of the animals were retrieved and processed for light microscopy and biochemical evaluation.

### 2.4. Immunohistological Analyses

**Figure 3:**
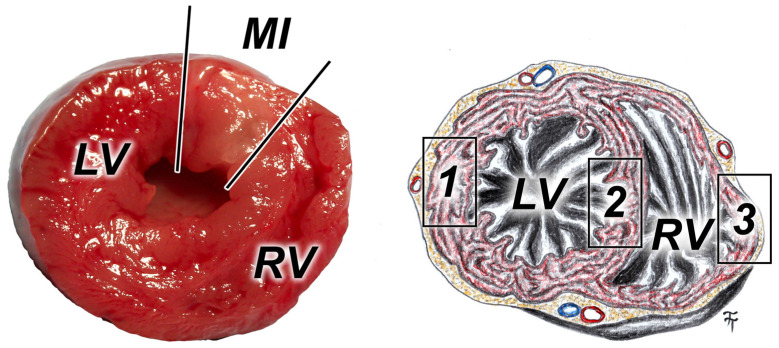
On the left—photo of a cross-section of the heart after its removal 14 days after myocardial infarction. On the right—an illustrative drawing of the cross-section of the heart with the locations of the immunohistological positivity evaluation (MI—myocardial infarction; LV—left ventricle; RV—right ventricle; 1–3—place of evaluation of *NCX1* expression in immunohistological samples).

The myocardium specimens were processed routinely for light microscopy: fixation, dehydration, embedding, cutting, and staining with hematoxylin-eosin and immunohistology for sodium/calcium exchanger 1 (*NCX1*) (Rabbit polyclonal Anti-*NCX1* antibody). The effort of antibody positivity was evaluated in three locations of the myocardium (free wall of the left ventricle, free wall of the right ventricle, and the interventricular septum) by two researchers semi-quantitatively on a scale from 0 to 4 (0—absent, 1—rare, 2—mild, 3—moderate, 4—significant presence of immunohistological positivity), as previously described [46,47] (Figure 3).

### 2.5. RNA Isolation and mRNA Gene Level Expression

All tissue samples were frozen in liquid nitrogen immediately after harvesting and stored in a freezer at −80 °C. Isolation of total RNA was performed according to the valid methodological protocol approved by the manufacturer of the RNeasy Mini Kit (Qiagene, Hilden, Germany). The concentration and quality control for purity in isolated RNA samples were assessed using the Qubit RNA assay kit (Thermo Fisher Scientific, Waltham, MA, USA). Changes in the expression level of mRNA for *NCX1* were detected. A specific reverse transcription assay using an M-MLV reverse transcriptase kit (Sigma-Aldrich, St. Louis, MO, USA) was performed. Experimental genes *NCX1* and control housekeeping genes (GAPDH) were amplified by 340 cycles using the appropriate specific primer sequences. Numerical quantification of changes in the expression of mRNA levels was evaluated by comparative quantification and ΔCt values using the Q Rotor-Gene software 2.1.0.9 (Qiagen, Hilden, Germany). After this normalization, the delta threshold cycle (ΔCT) values were used to determine the delta CT threshold cycle (ΔΔCT) and to obtain the relative amount of the mRNA to be determined using the formula for relative quantification (target gene 1) = 2 − ΔΔCT (target gene 1).

### 2.6. Statistical Analyses

Survival data are presented as absolute numbers and as the percentage of the group. Other data are presented as the average ± standard deviation. For statistical comparisons of the groups, the chi-squared test, analysis of variance followed by Tukey–Kramer multiple comparisons, and the Kruskal–Wallis test were used. For each test, significance was accepted at *p* < 0.05.

## 3. Results

**Figure 4:**
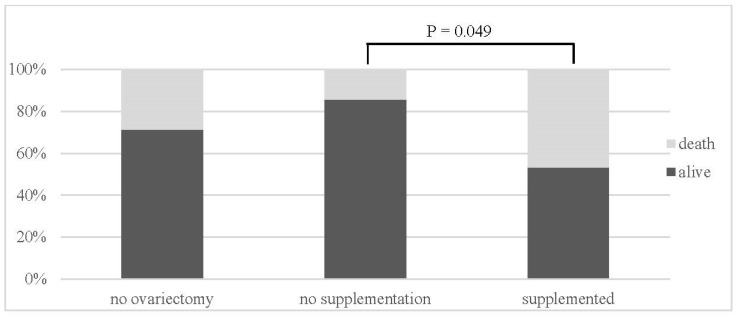
Survival in animal groups.

The survival rate after MI in all animals was recorded at 65%. A statistically significant higher survival was recorded in the OVX-N group (86%) in comparison with the OVX-S group (53%) (Table 1, Figure 4).

**Figure 5:**
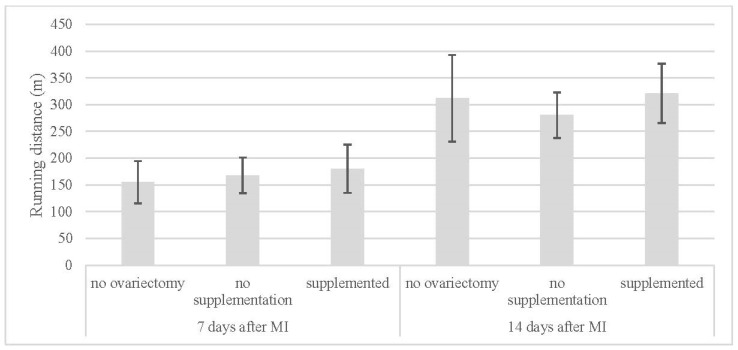
Running distance in groups.

The graded treadmill running test showed no differences between the subgroups 7 days after MI, nor was any statistically significant difference recorded between the subgroups 14 days after MI (Table 2, Figure 5). The running distance 14 days after MI was significantly higher statistically than the running distance 7 days after MI in every subgroup.

**Figure 6:**
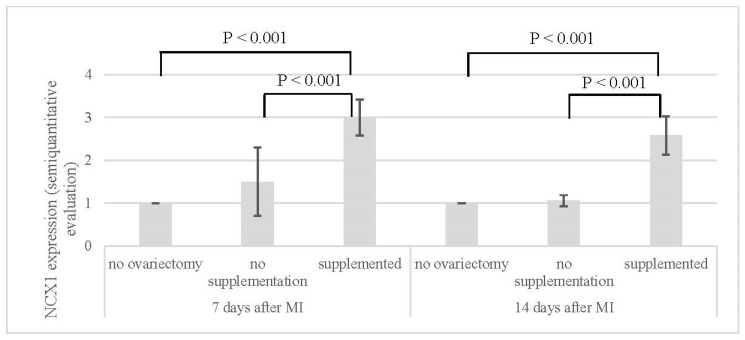
Semi-quantitative evaluation of *NCX1* expression in myocardium 7 and 14 days after MI.

**Figure 7:**
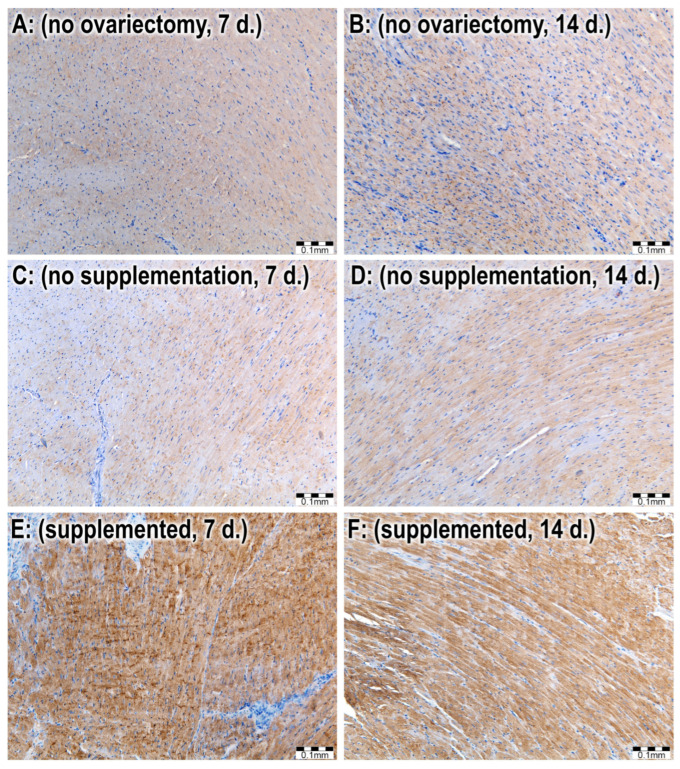
Immunohistology Rabbit polyclonal Anti-*NCX1* antibody 200×: (**A**)—subgroup NN_7; (**B**)—subgroup NN_14; (**C**)—subgroup OVX-N_7; (**D**)—subgroup OVX-N_14; (**E**)—subgroup OVX-S_7; (**F**)—subgroup OVX-S_14.

Statistical evaluation of *NCX1* expression showed statistically significant (*p* < 0.001) higher *NCX1* expression in the supplemented group in comparison with the group without ovariectomy and the group with ovariectomy but without estrogen supplementation. The same results were recorded in groups evaluated 7 days and 14 days after MI (Table 3, Figure 6 and Figure 7).

**Figure 8:**
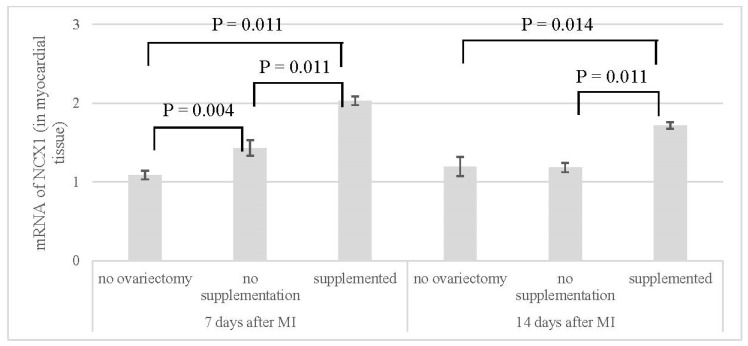
*NCX1* mRNA in myocardial tissue 7 and 14 days after MI.

**Figure 9:**
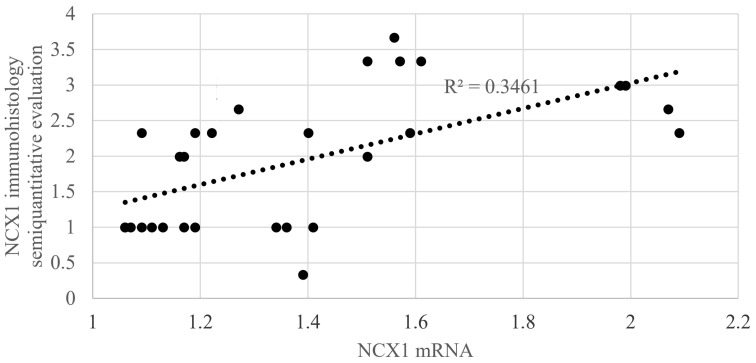
Correlation analysis of *NCX1* mRNA and *NCX1* immunohistology semi-quantitative evaluation.

Statistical evaluation of *NCX1* mRNA showed statistically significant (*p* < 0.001) higher *NCX1* mRNA expression in the supplemented group in comparison with the group without ovariectomy and the group with ovariectomy but without estrogen supplementation. The same results were recorded in groups evaluated 7 days and 14 days after MI. Statistically significant differences were also recorded between non-supplemented animals with ovariectomy in comparison with supplemented animals with ovariectomy. The difference between these two groups was recorded only 7 days after MI (Table 4, Figure 8). Correlation analysis of the *NCX1* immunohistology evaluation and *NCX1* mRNA showed r2 = 0.3461 (Figure 9).

## 4. Discussion

The sodium–calcium exchanger NCX1 is a membrane antiporter consists of a transmembrane domain with 10 transmembrane helices and a large intracellular regulatory domain which, in primary sequence, separates the transmembrane domain into two homologous halves (TMs 1–5 and TMs 6–10) [48]. In physiological conditions, the basic function of *NCX1* is to balance calcium homeostasis in cardiomyocytes by extruding cytoplasmic Ca^2+^ during myocyte repolarization [49,50,51]. During the repolarization phase, NCX1 facilitates the transport of Ca^2+^ out of the cell in exchange for three sodium cations. It is activated by calcium binding to CBD1, while calcium binding to CBD2 further prevents its inactivation. On the other hand, higher Na^+^ concentration or activation of the XIP domain inhibits NCX1 function [52]. The Na^+^-dependent inactivation occurs when three Na^+^ ions are bound to the inward-facing exchanger [48]. While sequestration by the phospholipid PIP2 inhibits the XIP domain, palmitoylation increases its sensitivity. Ultimately, non-palmitoylated NCX1 ensures enhanced transport of calcium ions across the cell membrane [52].

Inducing proper Ca^2+^ homeostasis during repolarization is essential for the correct course of the next systole of the heart. The action potential of electrical stimulation leads to the entry of a small amount of calcium cations into the cell, triggering a more massive release of Ca^2+^ from the sarcoplasmic reticulum. The binding of Ca^2+^ to myofilaments creates what is known as excitation–contraction coupling and activates the contractile mechanism [53]. The activity of NCX1 is not influenced solely by the presence of the ions themselves. Its activity is also affected by certain regulatory mechanisms that can either enhance or diminish its transport activity. One of these substances is calmodulin. Its activity is mediated by activation through calcium ions. The Ca^2+^–calmodulin complex then increases the effectiveness of NCX1, which subsequently reduces the concentration of Ca^2+^ [54]. *NCX1* overexpression is documented during human heart failure and in an animal model of heart hypertrophy and heart failure [55]. Moreover, Ca^2+^ enters into myocytes by specific channels that have been identified as a signal that triggers Ca^2+^-dependent expression of pro-hypertrophic genes [51,56,57]. During heart failure progression, overexpression of *NCX1* and TRPC3/*NCX1* interaction are compensatory mechanisms that improve systolic function [56,58]. Depressed *NCX1* activity during heart failure progression may accelerate contractile dysfunction [51,56]. Changes in NCX1 expression are significantly associated with stages of heart failure. While a slight increase in transcription during the early stages provides just enough Ca^2+^ for its increased demand, excessive expression leads to a decline in contractility and failure of compensatory mechanisms. Both α-adrenergic and β-adrenergic receptors play an important role in regulating calcium homeostasis by influencing the activity of NCX1 [59]. The smooth regulation and autoregulation of NCX1 efficacy during the cardiac cycle under varying metabolic demands of the organism play a critical role in calcium ion homeostasis and the stabilization of heart rhythm [60].

The expression of NCX1 in various tissues can be increased by different stimuli, such as angiotensin-II in blood vessels, tumor necrosis factor-α (TNF-α) in the airways, or endothelin-1 in the kidneys. The upregulation of NCX1 expression in the myocardium during chronic overload appears to have a more complex mechanism, in which histone deacetylases play a particularly important role. Inhibitors of histone deacetylases seem to be a potentially therapeutically effective approach to reduce excessive NCX1 expression and prevent its negative consequences leading to myocardial failure [59]. *NCX1* protein expression and *NCX1* mRNA vary differently in different periods after the hypertrophy heart model in mice, and the function of *NCX1* is affected by a variety of factors [51]. However, enhanced *NCX1* expression is known to cause a reduced release of Ca^2+^ from the sarcoplasmic reticulum, which leads to an increased risk of arrhythmias [51,55,56,61]. Thus, the *NCX1* inhibitor dichlorobenzamil hydrochloride (DCB) is an effective arrhythmias inhibitor [56]. On the other hand, in mice with *NCX1* function blocked, severe bradycardia, loss of the ability to maintain a stable heart rhythm, and various ventricular and supraventricular rhythm disorders are presented [61]. Published data suggest that while partial blocking of *NCX1* leads to stabilization of the heart rhythm, absolute blocking leads also to the arrhythmias presented [62,63,64,65].

**Figure 10:**
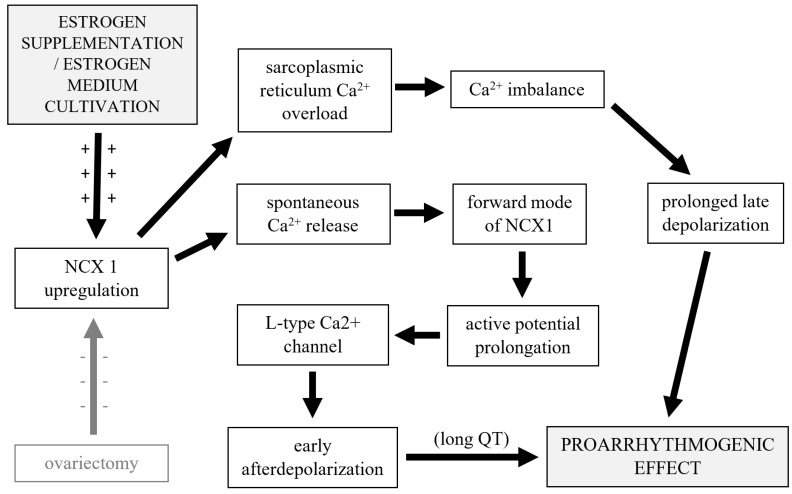
Potential pathophysiology mechanism of estrogen supplementation pro-arrhythmogenic effect (shadow pointer shows unsupported effect).

The pathological background of sex-dependent arrhythmia risk is particularly analogous with congenital or drug-induced long QT syndrome type 2 [66]. A relatively wide range of drugs leads to prolonged active depolarization and early afterdepolarization and causes drug-induced long QT syndrome, which can also cause lethal torsade de pointes [66]. Control of intracellular Ca^2+^ homeostasis is critical for cardiac function and myocyte survival [49,67]. Late afterdepolarization may be caused by an imbalance of Ca^2+^ influx and efflux, which is caused by sarcoplasmic reticulum Ca^2+^ overload and spontaneous release of Ca^2+^ [53,68,69]. This imbalance leads to activation of the forward mode of *NCX*, which causes action potential prolongation, reactivation of L-type Ca^2+^ channels, and triggers afterdepolarization [66]. In New Zealand, white rabbit ovariectomy caused a protective effect for drug-induced long QT syndrome-related arrhythmias [70]; however, 17-β-estradiol replacement therapy reversed this protective effect [66,71]. In our results, we also recorded a statistically non-significant decrease in mortality in the group of ovariectomized non-supplemented rats and a statistically significant increase in mortality after MI in rats treated with estrogen. According to the ECG recorded shortly after MI, mortality was affected mostly by the different arrhythmogenicity of the animals. A higher level of the L-type Ca^2+^ channel is associated with an elevation in *NCX* in the adult female heart [22,72]. Myocytes isolated from female hearts show higher levels of L-type Ca^2+^, and myocytes incubated with 17-β-estradiol show transcription upregulation of mRNA expression of *NCX* and the primary subunit of the L-type Ca^2+^ channel [6,22,73,74]. The increase in mortality caused by arrhythmogenicity after estrogen supplementation recorded in our study correlates with these data. The interesting thing is that this upregulation is regulated by only the α isoform of estrogen receptors [74]. Our findings of a higher density of *NCX* after estrogen supplementation correlate with the results of these authors. On the other hand, a lower density of *NCX* in ovariectomized rats in comparison with rats without ovariectomy was not recorded. Depressed *NCX1* expression after MI maintained the survival rate of these patients [55,75]. The function of *NCX1* also influences the diverse functions of myocytes, including excitation–contraction coupling and cell metabolism [49,76,77]. Despite these facts and the upregulation of *NCX1* in the supplemented group, we did not record any difference in the tolerance of physical activity after MI in the groups. The recorded results correlate with the pathophysiology mechanisms of estrogen supplementation presented by other authors. No effect of estrogen depression on *NCX1* mRNA and *NCX1* expression after ovariectomy was recorded (Figure 10).

The influence of NCX expression is not limited to the consequences for the cardiovascular system. Increased expression of NCX1 via the NFκB pathway triggers autophagic flux, which can affect the efficacy of certain drugs that work through this mechanism of action. If estrogen supplementation leads to increased expression in humans as well, it may influence the reduced sensitivity to bortezomib, which would negatively impact its effectiveness in the treatment of multiple myeloma [78]. In different tissues, the regulatory mechanisms influencing the function of NCX1 also partially vary. Its role is emphasized in the process of bone mineralization. Its activity in these tissues can be blocked by knocking out specific signaling pathways that contain, for example, Anoctamin-6, whose proper functioning is essential for the correct ossification of bone tissue [79].

Papp et al. also showed that myocardial cells derived from healthy human hearts show a similar response to very low estrogen concentration, with the upregulation of Ca^2+^ channels [66]. The authors reported that there is a close correspondence between experimental animal and human myocytes’ responses to estrogen.

## 5. Conclusions

The results suggest that estrogen supplementation therapy in rats leads to the upregulation of the sodium–calcium exchanger 1 (NCX1). It can be supposed that this results in an imbalance of Ca^2+^ homeostasis in myocardial cells. The results show that estrogen supplementation in rats causes increased susceptibility to cardiac rhythm disorders during myocardial infarction, as well as increased mortality as a consequence. This effect can be due to the upregulation of NCX1 and the resulting dysregulation of Ca^2+^ homeostasis. Published data from other authors suggest that a very similar mechanism may be expected in humans; moreover, at a very low estrogen dosage, too. This work is the first to document the potentially negative effect of estrogen therapy on myocardial infarction survival.

## Figures and Tables

**Table 1:** Survival in animal groups.

Group	Alive/Death	
no ovariectomy (NN)	10 (71%)/4 (29%)	
no supplementation (OVX-N)	12 (86%)/2 (14%)	*p* = 0.049 (OVX-N:OVX-S)
supplemented (OVX-S)	16 (53%)/14 (47%)	

**Table 2:** Running distance in subgroups at 7 and 14 days after myocardial infarction (MI—myocardial infarction, m—meters).

	Running Distance (m)	
	7 Days After MI	14 Days After MI
no ovariectomy (NN)	155 ± 40	312 ± 81
no supplementation (OVX-N)	168 ± 33	281 ± 43
supplemented (OVX-S)	180 ± 45	321 ± 56

**Table 3:** Semi-quantitative evaluation of *NCX1* in myocardium of animals.

	*NCX1* (Semi-Quantitative Evaluation)	
	7 Days After MI	14 Days After MI
no ovariectomy (NN)	1.0 ± 0.0	1.0 ± 0.0
no supplementation (OVX-N)	1.5 ± 0.8	1.1 ± 0.1
supplemented (OVX-S)	3.0 ± 0.4	2.6 ± 0.4

**Table 4:** mRNA of *NCX1* in myocardial tissue of animals.

	mRNA of *NCX1*	
	7 Days After MI	14 Days After MI
no ovariectomy (NN)	1.09 ± 0.06	1.20 ± 0.12
no supplementation (OVX-N)	1.43 ± 0.10	1.18 ± 0.06
supplemented (OVX-S)	2.03 ± 0.06	1.72 ± 0.04

## Data Availability

The data presented in this study are available on request from the corresponding author due to ongoing further research. The provision of data will be considered on an individual basis.
